# Activin A directly impairs human cardiomyocyte contractile function indicating a potential role in heart failure development

**DOI:** 10.3389/fcvm.2022.1038114

**Published:** 2022-11-10

**Authors:** Scott MacDonnell, Jake Megna, Qin Ruan, Olivia Zhu, Gabor Halasz, Dan Jasewicz, Kristi Powers, Hock E, Maria del Pilar Molina-Portela, Ximei Jin, Dongqin Zhang, Justin Torello, Nicole T. Feric, Michael P. Graziano, Akshay Shekhar, Michael E. Dunn, David Glass, Lori Morton

**Affiliations:** ^1^Regeneron Pharmaceuticals, Tarrytown, NY, United States; ^2^TARA Biosystems Inc., Alexandria Center for Life Sciences, New York, NY, United States

**Keywords:** hiPSC cardiomyocyte, Activin A, SERCA2a, myocardial contractility, heart failure

## Abstract

Activin A has been linked to cardiac dysfunction in aging and disease, with elevated circulating levels found in patients with hypertension, atherosclerosis, and heart failure. Here, we investigated whether Activin A directly impairs cardiomyocyte (CM) contractile function and kinetics utilizing cell, tissue, and animal models. Hydrodynamic gene delivery-mediated overexpression of Activin A in wild-type mice was sufficient to impair cardiac function, and resulted in increased cardiac stress markers (N-terminal pro-atrial natriuretic peptide) and cardiac atrophy. In human-induced pluripotent stem cell-derived (hiPSC) CMs, Activin A caused increased phosphorylation of SMAD2/3 and significantly upregulated *SERPINE1* and *FSTL3* (markers of SMAD2/3 activation and activin signaling, respectively). Activin A signaling in hiPSC-CMs resulted in impaired contractility, prolonged relaxation kinetics, and spontaneous beating in a dose-dependent manner. To identify the cardiac cellular source of Activin A, inflammatory cytokines were applied to human cardiac fibroblasts. Interleukin -1β induced a strong upregulation of Activin A. Mechanistically, we observed that Activin A-treated hiPSC-CMs exhibited impaired diastolic calcium handling with reduced expression of calcium regulatory genes (*SERCA2*, *RYR2*, *CACNB2*). Importantly, when Activin A was inhibited with an anti-Activin A antibody, maladaptive calcium handling and CM contractile dysfunction were abrogated. Therefore, inflammatory cytokines may play a key role by acting on cardiac fibroblasts, causing local upregulation of Activin A that directly acts on CMs to impair contractility. These findings demonstrate that Activin A acts directly on CMs, which may contribute to the cardiac dysfunction seen in aging populations and in patients with heart failure.

## Introduction

Activin A is a member of the transforming growth factor-β (TGF-β) superfamily and consists of a homodimer of two βA subunits (1). It was initially characterized as an inducer of follicle-stimulating hormone secretion but is now known to be involved in many critical biological processes, including embryonic development, cellular differentiation, hematopoiesis, tissue repair, and fibrosis (2–6). Functions of Activin A are mediated by activation of the SMAD2/3 pathway by binding to type I and type II activin receptors (7). Type I receptors include activin receptor type 1A (ALK2), activin receptor type 1B (ALK4), and activin receptor type 1C (ALK7), in humans. Activin receptor type 2A and activin receptor type 2B are type II receptors (8).

Emerging evidence suggests that Activin A also has an effect on the cardiovascular system, with elevated levels found in patients with pulmonary hypertension, atherosclerosis, and more recently coronavirus disease 2019 (COVID-19) (9–13). In heart failure, serum levels of Activin A are elevated and positively correlate both with severity of disease and age-dependent cardiac dysfunction (14, 15). Based on these findings, several labs have worked to explore potential mechanism(s) by which Activin A contributes to the pathogenesis of heart failure. Using cultured neonatal rat cardiomyocytes (CMs), Activin A caused a marked increase in expression of cardiac injury markers (atrial natriuretic peptide, and brain natriuretic peptide), markers of extracellular matrix remodeling (14), and reduced expression of sarco/endoplasmic reticulum Ca^2+^-ATPase 2a (SERCA2a) RNA and protein (15). Extending these findings *in vivo*, overexpression of Activin A in mice triggered cardiac activin receptor type II-mediated signaling in the heart, causing impaired systolic and diastolic cardiac function (15). Importantly, Roh et al. demonstrated that blocking activin receptors or activin receptor ligands prevented pressure overload-induced cardiac dysfunction, suggesting that Activin was a central contributor to the cardiac dysfunction observed in that model (15). Mechanistically, depletion of SERCA2a was observed in both neonatal rat CMs and in mice exposed to high levels of Activin A. These reductions in SERCA2a are plausible explanations for the reduced contractile function observed (15, 16). Despite these important advances in the understanding of how Activin A contributes to cardiac dysfunction, there are no functional data to provide clear evidence that Activin A has a direct impact on human CMs.

Human-induced pluripotent stem cells (hiPSCs) are a key tool for studying physiology and disease at the cellular level. The pluripotent nature of these cells allows for differentiation into specific cell types of interest that may not be readily available for research. Differentiation into human CMs provides researchers the opportunity to culture, treat, challenge, and monitor cell function over time, characteristics that are not feasible with adult rodent or human CMs (17). While hiPSC-CMs provide a model system for exploring disease biology, in 2D cultures they display disorganized sarcomere structure, express developmental genes, and are not cultured under a defined load. Therefore, when using hiPSC-CMs to define a phenotype, it is important not to rely solely on 2D cultures (17, 18). In this study, we characterize the direct impact of Activin A on murine ventricular function, hiPSC-CMs, and human engineered cardiac tissues (HECTs). We propose a mechanism by which inflammatory signaling drives cardiac Activin A expression and contributes to CM dysfunction in patients with heart failure.

## Materials and methods

### Tissue culture

For the culture of hiPSC-CMs, tissue culture vessels were pre-coated with 10 μg/mL fibronectin (Thermo Fisher Scientific, Waltham, MA, USA) for 1 h at 37°C. hiPSC-CMs (iCell Cardiomyocytes^2^; Fujifilm Cellular Dynamics, Madison, WI, USA) were stored, thawed, and plated according to the manufacturer’s instructions. Briefly, cells were flash thawed (37°C, 3 min) and slowly diluted in plating medium. Day 0 is the timepoint of differentiated hiPSC-CM plating. Differentiation is complete as the iCell^2^ cardiomyocytes follow a 17–20-day differentiation protocol performed prior to cell shipment. The iCell^2^ cardiomyocytes arrived cryopreserved, and are genetically purified to approximately 98% cTnT + cells. For gene expression analysis and phosphorylation assays, 5 × 10^5^ cells were plated per well of a 12-well plate. For impedance, electrophysiology, and calcium transient assays, cells were plated in 96-well plates at a density of 5 × 10^4^ cells per well. Cells were maintained in a humidified 37°C incubator with 5% CO_2_, with media changed every 48 h. Cells were maintained in culture until a synchronous, beating monolayer of cells formed (∼10–14 days).

Primary human cardiac fibroblasts (HCFs; PromoCell, Heidelberg, Germany) were plated in 24-well plates and grown (37°C, 5% CO_2_) in medium containing 10% serum, until 80% confluent. Cells were then incubated with or without 1 nM recombinant human interleukin (IL)-1β, oncostatin M, or IL-6 (all purchased from R&D Systems, Minneapolis, MD, USA) in Dulbecco’s Modified Eagle Medium + 0.1% bovine serum albumin for 24 h.

### Western blot analysis

Cells were exposed to 1 nM Activin A (R&D Systems) for 30 min in the presence or absence of anti–Activin A (10 nM REGN2477) or isotype control (10 nM REGN1945) monoclonal antibodies (Regeneron Pharmaceuticals, Tarrytown, NY, USA) (19, 20). Cells were washed twice with cold phosphate-buffered saline and lysed using RIPA Lysis and Extraction Buffer (Thermo Fisher Scientific) supplemented with Halt™ Protease and Phosphatase Inhibitor Cocktail (Thermo Fisher Scientific). Lysates were centrifuged (14,000 × *g*, 15 min), and the total protein was quantified using the Pierce BCA Protein Quantitation Kit (Thermo Fisher Scientific). Protein detection in cell lysates was performed under reducing conditions using a 12–230 kDa Separation Module for the capillary electrophoresis™ Wes system (ProteinSimple, San Jose, CA, USA), according to the manufacturer’s instructions. Protein samples were diluted with 5 times reducing buffer to a final concentration of 0.5 mg/mL, denatured (5 min, 95°C), and placed on ice. Cartridge plates were assembled, spun (1,000 × *g*, 5 min), and placed into the Wes™ instrument. Primary antibodies were obtained from Cell Signaling Technologies (Danvers, MA, USA). Phospho-SMAD2(Ser465/467)/SMAD3(Ser423/425) was diluted 1:50, SMAD2/3 was diluted 1:50, and GAPDH was diluted 1:100. The Anti-Rabbit Detection Module (ProteinSimple) consisted of anti-rabbit secondary antibody, a streptavidin-horseradish peroxidase conjugate, and chemiluminescent detection reagents. Protein detection was analyzed using Compass software (ProteinSimple), which quantified areas under the curves and height for peak chemiluminescent signals from the proteins of interest.

### RNA isolation and cDNA synthesis

hiPSC-CMs were exposed to Activin A (R&D Systems) acutely (1 nM for 24 h) or chronically (1 nM every 48 h for 6 doses), and gene expression was analyzed. RNA was isolated using an RNeasy Mini Kit (Qiagen, Germantown, MD, USA) according to the manufacturer’s instructions. RNA concentration and quality were assessed using a NanoDrop Spectrophotometer (Thermo Fisher Scientific). cDNA was synthesized using the Maxima™ H Minus cDNA Synthesis Master Mix (Thermo Fisher Scientific).

For mouse gene expression analyses, tissues were homogenized in TRIzol (Thermo Fisher Scientific) with chloroform for phase separation. Total RNA was then purified from the aqueous phase using a MagMAX™-96 for Microarrays Total RNA Isolation Kit (Thermo Fisher Scientific) according to the manufacturer’s instructions. Genomic DNA was removed using an RNase-Free DNase Set (Qiagen, Germantown, MD, USA). cDNA synthesis was performed using the SuperScript^®^ VILO™ Master Mix (Thermo Fisher Scientific) and quantified using NanoDrop (Thermo Fisher Scientific).

### Bulk RNA sequencing

Strand-specific RNA-seq libraries were prepared from 500 ng RNA using a KAPA stranded mRNA-Seq Kit (KAPA Biosystems, Wilmington, MA, USA). Twelve-cycle polymerase chain reaction (PCR) was performed to amplify libraries. Sequencing was performed on an Illumina HiSeq^®^ 2500 (Illumina, San Diego, CA, USA), using a multiplexed paired-read run with 33 cycles. Raw sequence data were converted to FASTQ format *via* Illumina Casava 1.8.2. Reads were decoded based on their barcodes, and quality was evaluated with FastQC. Reads were mapped to the human genome (NCBI B37.3) and a University of California, Santa Cruz gene model using ArrayStudio^®^ software (OmicSoft, Cary, NC, USA), allowing 2 mismatches. Reads mapped to the sense-strand exons of a gene were summed at the gene level.

### Bulk RNA-seq differential analysis

Differential gene expression analysis was carried out with DESeq2 v1.30.0 (Bioconductor software) (21). Counts at the gene level as summarized by ArrayStudio^®^ software were used as input after rounding. Genes were pre-filtered for a minimum of 10 reads. Size factors were estimated per sample using a median-of-ratios method to account for inter-sample sequencing depth variations. Hypothesis testing was carried out using the Wald test, with multiple testing correction by the Benjamini and Hochberg method. Significance was defined as adjusted *P* < 0.05 and fold change > 1.5.

### Gene set enrichment analysis

All genes were sorted using –log10(pval)*FC from DESeq2 analysis (regardless of significance). The pre-sorted gene lists for each comparison were used as input into Gene Set Enrichment Analysis (GSEA) using the fast gene set enrichment analysis R package v1.16.0 (Bioconductor software) (22). Gene sets tested were obtained from the Molecular Signature Database c2.cp.reactome.v7.1 collection (23).

### Gene expression analysis by RT-qPCR

Polymerase chain reactions (20 μL total) contained 10 μL of 2 × TaqMan Gene Expression Master Mix (Thermo Fisher Scientific), 1 μL of a 20 × TaqMan probe (Supplementary Table 1; Thermo Fisher Scientific), 5 μL (10 ng) of cDNA, and 4 μL of water, and were run on a QuantStudio™ 3 Real-Time PCR System (Thermo Fisher Scientific). Thermocycler settings were as follows: 95°C for 15 min, then 40 cycles of 95°C, 15 s followed by 60°C, 60 s. Amplification plots were generated by QuantStudio 3 instrument software, and resulting cycle threshold (Ct) values were derived. *GAPDH* was used as an endogenous control. Analysis of gene expression in mouse tissues was performed using a SensiFAST Probe Lo-ROX Kit (Meridian Bioscience, Memphis, TN, USA) with the QuantStudio 12K Flex Real-Time PCR System (Thermo Fisher Scientific). *GAPDH* was used as an endogenous control gene to normalize any cDNA input differences. The delta-delta Ct (2^–ΔΔCt^) method (24) was used to calculate relative fold change in gene expression for all quantitative reverse transcription PCR (RT-qPCR) analyses.

### Impedance and electrophysiological characterization of hiPSC-CMs

Following an initial dose-response assessment of cells exposed to titrated Activin A (R&D Systems), a 1 nM dose was chosen for subsequent experiments unless otherwise stated. For impedance, electrophysiology, and calcium assays, chronic exposure to Activin A was performed as shown in the presence or absence of an anti–Activin A (REGN2476) or an isotype control antibody (REGN1945) (Regeneron Pharmaceuticals Inc.) (25). Contractility and electrophysiology of hiPSC-CMs were characterized using CardioExcyte 96 (Nanion Technologies, Munich, Germany), a hybrid system that simultaneously records the impedance and extracellular field potential (EFP) of a beating monolayer of CMs in a label-free environment under physiological culture conditions. In this study, hiPSC-CMs were plated on electrode-containing 96-well plates (NSP-96; Nanion Technologies) and recorded for 30 s every 4 h. Impedance and EFP data were analyzed using DataControl 96 software (Nanion Technologies). All beating inflections recorded during the 30-s interval were sectioned and averaged to derive a “mean beat.” The following electrical pacing parameters were used during impedance recording: pace frequency, 1 Hz; burst amplitude, 1 volt; burst frequency, 1 kHz; burst length, 2 ms. The amplitude (peak-to-trough signal), fall/rise time (time from 90 to 10% amplitude and vice versa), upstroke/relaxation velocity (maximal positive and negative slopes), and overall coverage of the electrode through the base impedance (steady component of the impedance magnitude) were characterized from the profile of each mean beat (26, 27). For EFP recording, transient electrical activity outside of the cell was measured, and mean beats were subsequently inversed to resemble the action potential of CMs. Amplitude, downstroke velocity (maximal slope during depolarization), and field potential duration (the time between the first deflection for depolarization and the maximum of the repolarization curve) were characterized for each mean beat.

### Calcium transient measurements in hiPSC-CMs

Calcium transients were assessed using an EarlyTox Cardiotoxicity Kit (Molecular Devices, San Jose, CA, USA) (28). Calcium dye loading was performed according to the manufacturer’s instructions. EarlyTox calcium dye was resuspended in the supplied buffer and added to the cells in a 1:1 ratio with the CM maintenance media. The plate was incubated for 2 h (37°C, 5% CO_2_) before recording calcium transients for 2 min at 37°C on the FLIPR Tetra System (Molecular Devices), using the following parameters: excitation, 470–495 nM; emission, 515–575 nM; exposure time, 50 ms; light-emitting diode intensity, 50%; and interval time, 100 ms. Calcium traces were produced and analyzed using SoftMax Pro Software (Molecular Devices).

### Generation of HECTs and image-based contractility measurements

Three-dimensional HECTs suspended between poly[octamethylene maleate (anhydride) citrate] wires were prepared from hiPSC-CMs and human ventricular cardiac fibroblasts (Lonza, Allendale, NJ, USA), and image-based contractility measurements were generated using the Biowire II platform (TARA Biosystems, Inc.), as described previously (29, 30). HECTs were assessed for automaticity (i. e., spontaneous beat rate), force-frequency relationship (FFR; active force at 1–4 Hz), and post-rest potentiation. HECTs with minimal spontaneous activity that exhibited a positive FFR and post-rest potentiation were used for compound testing. HECTs were incubated for 30 min in an environmental chamber (37°C, 5% CO_2_) before video recording under field stimulation at 1 Hz for 30 s followed by 30 s without field stimulation. Culture media were then removed and replaced with fresh media containing the test compound or vehicle. Arrhythmic activity was determined from contractile measurements acquired every 48 h followed by reapplication of the test compound or vehicle. Contractility videos were analyzed using custom analysis software. Twitch amplitude (active force), log2 transformation of twitch amplitude, twitch duration (twitch width at half amplitude), time to twitch amplitude (10% twitch height to amplitude), time from twitch amplitude (amplitude to 10% twitch height), maximum contraction and relaxation slopes, and percent arrhythmic beats (doublet beats/total beats x 100) were characterized for each 30-s video acquired with field stimulation. The spontaneous beat rate was calculated from each of the 30-s videos acquired without field stimulation (total beats/30 s).

### Animals

Male C57BL/6N mice aged ∼12 weeks and weighing 22–28 g (Taconic Biosciences, Rensselaer, NY, USA) were acclimated for a minimum of 7 days prior to experimentation. Mice were co-housed in polycarbonate, solid-bottom cages in a temperature-controlled environment (22 ± 2°C) with an approximate 12-h light-dark cycle and with access to research diets of standard pellet chow and reverse osmosis–filtered water. Mice were anesthetized with a mixture of 2% isoflurane in 100% oxygen before surgical procedures. All animal procedures and protocols described in this work were approved by Regeneron Pharmaceuticals, Inc., are in accordance with state and federal guidelines, and are aligned with regulations set forth by Regeneron Pharmaceuticals, Inc.’s Institutional Animal Care and Use Committee.

### Hydrodynamic delivery of an Activin A expression construct

cDNA-encoding Activin A was generated by PCR from a plasmid template harboring untagged Activin A (RefSeq accession number NM_002192.4) using the following primers: forward, 5′-TCCCCCACCCCAGGATCCGAGGG GCCA-3′ and reverse, 5′-GCACCTCCTCACACCCACGAGTAT CCGCCGGCGCCTAGGATCTAG-3′. The resulting cDNA was cloned into a mammalian expression vector pRG977 and confirmed by DNA sequencing. On study day 0, mice (*n* = 20) were stratified to groups based on body weight. Mice were injected *via* tail vein with 2.5 μg plasmid in sterile saline at 10% of body weight. Serum was collected to assess Activin A expression and biomarkers. At study termination (14 days post-hydrodynamic delivery [HDD]), terminal body weight was acquired and immediately followed by administration of a terminal dose of ketamine/xylazine and exsanguination *via* the abdominal aorta. The heart was resected and weighed, and the right and left atria and ventricles were dissected and placed immediately into individual tubes containing RNAlater (Thermo Fisher Scientific). The tibia was removed and measured using a hand caliper.

### Pressure–volume loop acquisition

Mice (*n* = 26) that underwent hydrodynamic delivery of Activin A for 14 days were placed in a supine position on a warm surface with external heating lamps to maintain normal body temperature (35.5–37.5°C). Endotracheal intubation was performed, and mice were ventilated (Harvard MiniVent with PEEP attachment) at 135 breaths/minute and tidal volume 0.2 mL. An incision was made in the upper abdomen above the xiphoid toward the anterior. The skin was then separated from the chest wall *via* blunt dissection, and a small incision was made above the apex of the heart between the third and fourth intercostal space. A small saline-dipped swab was placed through the incision. Using the swab to protect the heart and lungs, a cauterized cut was made through the ribs and sternum to the opposite side of the chest to expose the apex of the heart and the inferior vena cava (IVC). Forceps were used to remove the pericardium. The apex of the left ventricle was punctured with a 25-gauge needle, and a 1.2 F tetrapolar admittance catheter (Transonic Systems, Ithaca, MY, USA) attached to an ADV500 pressure–volume measurement system (Transonic Systems) and PowerLab 16/35 interface (ADInstruments, Colorado Springs, CO, USA) was inserted. Baseline recording was conducted for 5 min before making 3 IVC occlusions in succession. Data were analyzed using LabScribe (iWorx, Dover NH, USA).

### ELISA

Activin A levels were assessed using the Activin A Quantikine enzyme-linked immunosorbet assay (ELISA) kit (R&D Systems), and N-terminal pro-atrial natriuretic peptide (NT-proANP) levels were assessed using an NT-proANP ELISA (Biomedica Medizinprodukte, Vienna, Austria) according to the manufacturer’s instructions.

### Statistical analyses

For *in vitro* parameters, data are expressed as mean ± standard deviation. One-way analysis of variance with Tukey’s *post hoc* analysis was used to determine significance. For all *in vivo* parameters evaluated, statistical analyses were performed using GraphPad Prism (GraphPad Software, San Diego, CA, USA). Data are expressed as mean ± standard error of the mean. Statistical analyses were performed using an unpaired 2-tailed t-test. *P* values ≤ 0.05 were considered indicative of statistical significance.

## Results

### Elevated circulating Activin A results in cardiac dysfunction in the murine heart

**FIGURE 1:**
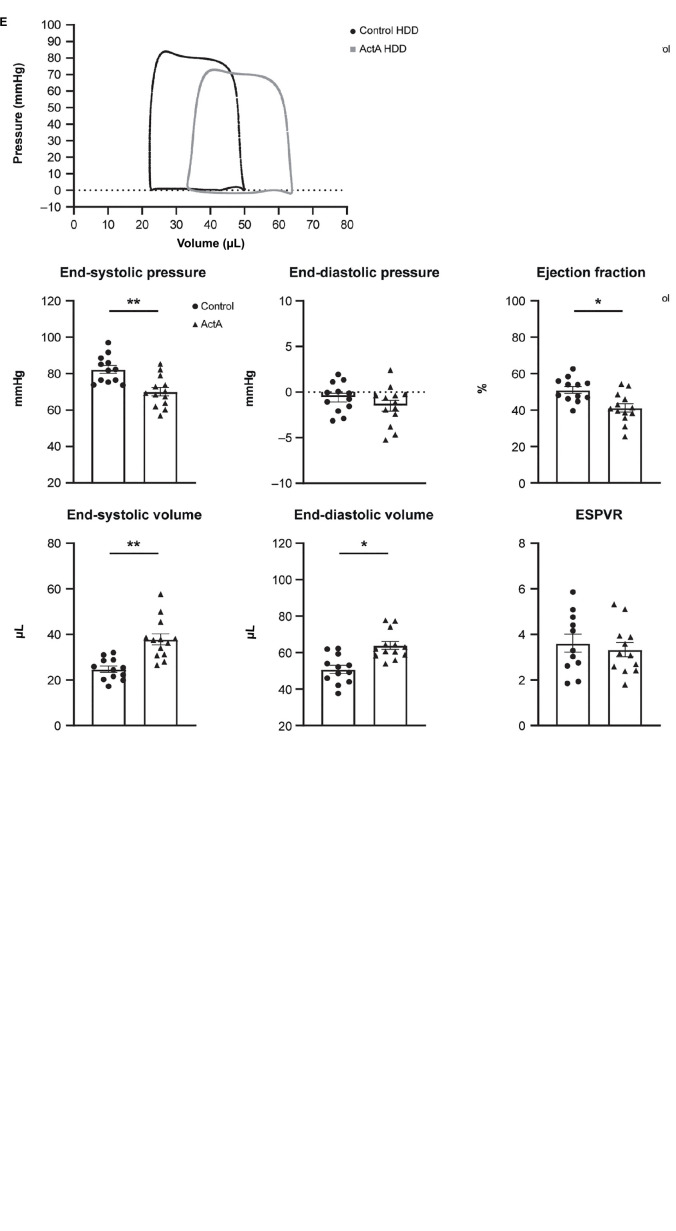
Activin A overexpression in mice produced cardiac dysfunction. Comparison of physical and molecular characteristics in control and Activin A–treated mice. **(A)** *In vivo* circulating levels of Activin A. **(B)** Heart-to-body weight ratio. **(C)** Circulating NT-proANP. **(D)** Gene expression in LV + S tissue. **(E)** Pressure-volume loop analyses. Statistical analyses were performed using an unpaired two-tailed t-test. *P < 0.05, **P < 0.01. ActA, Activin A; ESPVR, end systolic pressure–volume relationship; HDD, hydrodynamic delivery; LV + S, left ventricle and septum; NT-proANP, N-terminal pro-atrial natriuretic peptide.

To investigate whether Activin A is sufficient to alter global heart function, we challenged wild-type mice with a mammalian expression vector encoding Activin A (Activin A HDD) and assessed cardiac function using pressure–volume loop analysis. Levels of circulating Activin A were ∼80 times higher in mice injected with Activin A HDD compared with mice injected with control vector (*P* < 0.01; Figure 1A). In mice exposed to high levels of Activin A for 14 days, the heart-to-body weight ratio was ∼9% lower (*P* < 0.01), and the heart weight-to-tibia length ratio was ∼15% lower (*P* < 0.01) (Figure 1B). Levels of circulating NT-proANP, a biomarker of cardiac wall stress, were > 1.7 times higher (*P* < 0.01) in mice injected with Activin A HDD (Figure 1C).

Significant increases in expression of *Fstl3* (∼1.5-fold [*P* < 0.05]), *Nppa* (∼1.8-fold [*P* < 0.05]), and *Myh7* (∼1.4-fold [*P* < 0.05]) in left ventricle and septum tissue compared with control were seen with Activin A, while no significant changes were observed in Activin A-induced expression of *Nppb*, *Myh6*, *Tgf*-β*1*, and *Gdf15* (Figure 1D).

Pressure–volume loop analysis revealed that Activin A caused significant decreases in end-systolic pressure (∼15%, *P* < 0.01) and ejection fraction (∼19%, *P* < 0.05), and caused significant increases in end-systolic and end-diastolic volumes (∼53%, *P* < 0.01 and ∼26%, *P* < 0.05, respectively) (Figure 1E). Although Activin A HDD resulted in circulating Activin A concentrations (mean value: 4.7 ng/mL) that were higher than those observed in human heart failure (<1 ng/mL observed in human heart failure patients) (14), strikingly, Activin A was sufficient to induce impaired systolic and diastolic cardiac function in otherwise healthy young mice.

### Activin A increases SMAD phosphorylation and expression of SMAD2/3 target genes in hiPSC-CMs

**FIGURE 2:**
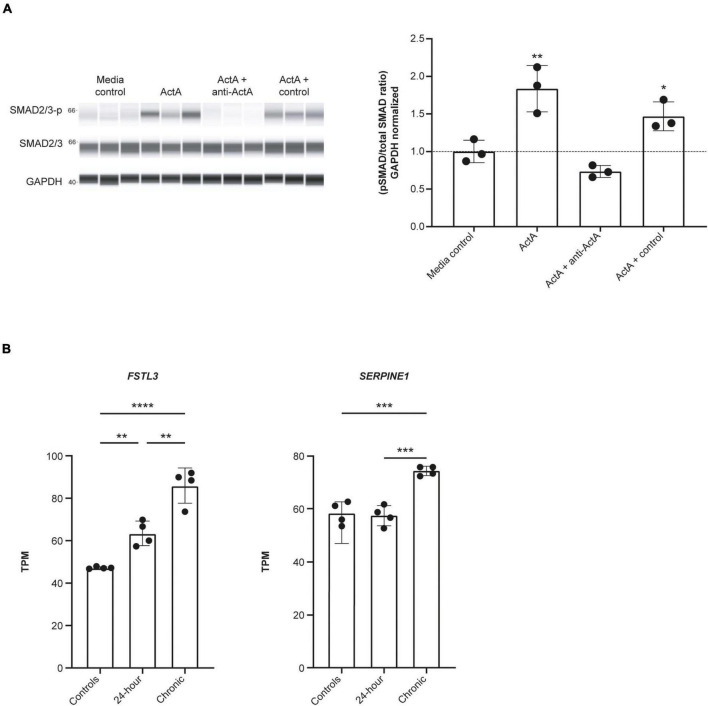
Activin A induces SMAD3 phosphorylation and downstream signaling in hiPSC-CMs. **(A)** Automated western blot data for the effect of Activin A plus anti–Activin A antibody or an isotype control on SMAD2/3 phosphorylation. **(B)** RNA sequencing data for expression of *FSTL3*, and *SERPINE1* (PAI1) expression following exposure to 1 nM Activin A. One-way ANOVA was used to determine significance. *P < 0.05, **P < 0.01, ***P < 0.001, ****P < 0.0001. ActA, Activin A; hiPSC-CM, human-induced pluripotent stem cell-derived cardiomyocyte; GAPDH, glyceraldehyde 3-phosphate dehydrogenase; TPM, transcripts per million.

We next explored whether Activin A could directly drive downstream signaling in human CMs. SMAD2/3 phosphorylation was significantly increased by 83% in hiPSC-CMs exposed to 1 nM Activin A (*P* < 0.01) for 30 min compared with control (Figure 2A). This increase in SMAD phosphorylation was blocked by the anti–Activin A antibody but not by an isotype control (Figure 2A).

RNA sequencing was used to determine the effect of acute (24 h) and chronic (6 repeated doses) Activin A exposure on expression of genes directly involved in activin signaling, including receptors, ligands, and SMAD proteins. With chronic exposure, expression of the genes encoding ALK2 (*ACVR1*) and bone morphogenic protein receptor type 2 (*BMPR2*) increased by 46% and 28%, respectively, from baseline following chronic exposure to Activin A (Table 1). Similarly, expression of *SMAD3* and *TGF*-β*1* increased by 22% and 40%, respectively. Expression of downstream SMAD2/3 target genes was also higher compared with control cells following chronic Activin A exposure, including *FSTL3* (∼83%, *P* < 0.0001) and *SERPINE1* (∼27%, *P* < 0.001), indicating that activin-dependent SMAD signaling was intact in these hiPSC-CMs (Figure 2B). Interestingly, acute Activin A exposure caused a ∼33% increase in expression of *FSTL3* (*P* < 0.01), but had no significant effect on expression of *SERPINE1*, while chronic treatment induced upregulation of both *FSTL3* and *SERPINE1* (Figure 2B).

**TABLE 1:** Effect of Activin A on expression of genes involved in activin signaling.

		Gene expression (TPM)		
		
	Gene (protein)	Baseline	Activin A (24 h)	Activin A (chronic)
Receptors	*ACTRIIA* (ACVR2A)	12.3	12.0	12.5
	*ACTRIIB* (ACVR2B)	14.3	13.5	13.0
	*ACVR1* (ALK2)	42.1	55.0	61.3
	*ACVR1B* (ALK4)	34.0	37.8	37.7
	*ACVR1C* (ALK7)	0.2	0.1	0.1
	*BMPR2*	88.6	107.0	113.0
	*BMPR1A* (ALK3)	85.0	85.0	86.0
	*BMPR1B* (ALK6)	0.1	0.2	0.2
SMADs	*SMAD2*	43.9	44.2	45.7
	*SMAD3*	65.0	76.0	79.0
	*SMAD4*	45.5	48.9	45.4
	*SMAD1*	21.8	21.8	19.8
	*SMAD5*	30.5	30.4	31.2
Ligands	*INHBA*	19.8	16.5	24.6
	*INHBB*	0.1	0.1	0.1
	*INHBC*	0.1	0.2	0.1
	*GDF11*	22.7	20.7	23.8
	*MSTN* (GDF8)	0.1	0.1	0.1
TGF-β ligand	*TGF*-β*1*	69.8	83.3	97.4
	*TGF*-β*2*	17.5	14.8	17.4
	*TGF*-β*3*	5.3	5.4	5.9

ActRII, activin receptor type 2; ACVR, activin A receptor type 2A; ALK2, activin receptor type 1; ALK3, activin receptor-like kinase; ALK4, activin receptor type 1B; ALK6, bone morphogenetic protein receptor type-1B; ALK7, activin receptor type 1C; BMPR, bone morphogenic protein receptor; GDF8, growth differentiation factor 8; MSTN, myostatin; TGF-β, transforming growth factor-β; TPM, transcripts per million.

### Activin A impairs hiPSC-CM contractile function

**FIGURE 3:**
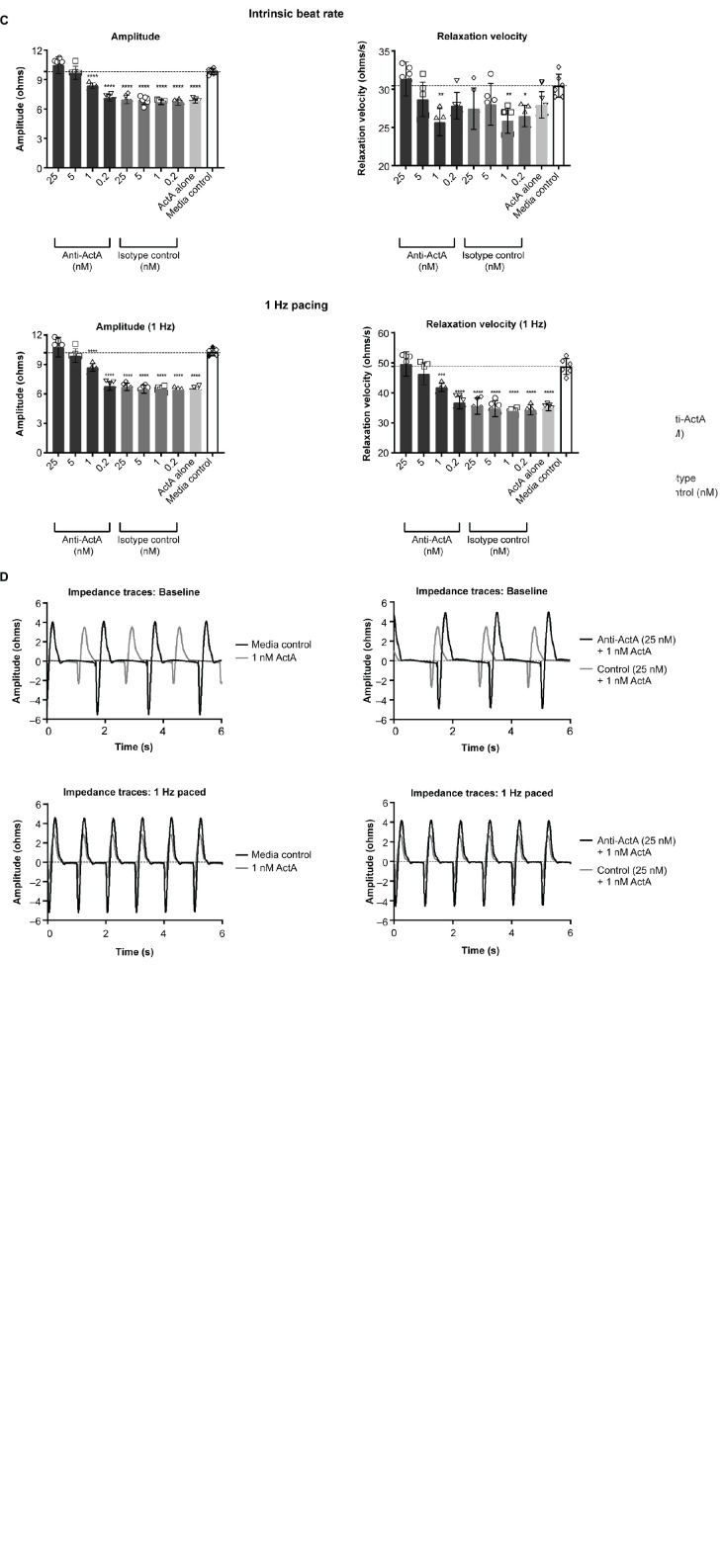
Activin A exposure time course on hiPSC-CM function. **(A)** The effect of different concentrations of Activin A on hiPSC-CM contractile impedance amplitude. **(B,C)** The impact of Activin A inhibition on Activin A–induced contractile dysfunction in hiPSC-CMs: dose-dependent effect on contractile impedance amplitude **(B)** and contractile parameters at both intrinsic and normalized beat rate **(C)**. **(D)** Representative traces at both intrinsic beat rate and paced at 1 Hz hiPSC-CM, induced pluripotent stem cell-derived CM. Vertical lines indicate media changes in panels **(A,B)**. One-way ANOVA was used to determine significance. *P < 0.05, **P < 0.01, ***P < 0.001, ****P < 0.0001. ActA, Activin A; CM, cardiomyocyte; hiPSC-CM, human-induced pluripotent stem cell-derived cardiomyocyte.

To study the direct consequence of engaged Activin A signaling on hiPSC-CM contractile function, we conducted Activin A dose-escalation studies and continuously measured contractile dynamics for 21 days post-exposure. A dose-dependent decrease in contractile impedance amplitude was observed in hiPSC-CMs after exposure to Activin A (Figure 3A). From day 10 (baseline) to day 20, contractile amplitude decreased by 63% with 100 nM Activin A (11.6 ± 0.264 ohms to 4.33 ± 0.726 ohms), 62% with 10 nM Activin A (12.6 ± 0.334 ohms to 4.76 ± 0.117 ohms), 53% with 1 nM Activin A (11.7 ± 0.142 ohms to 5.46 ± 0.126 ohms), and 32% with 0.1 nM Activin A (11.9 ± 0.177 ohms to 8.06 ± 0.336 ohms), compared with a 21% reduction in control media (12.2 ± 0.175 ohms to 9.65 ± 0.222 ohms). The Activin A–induced decrease in contractile impedance amplitude was prevented by 25 nM anti–Activin A monoclonal antibody (10.50 ± 0.87 vs 6.97 ± 0.43 ohms in cells exposed to Activin A plus isotype control antibody; *P* < 0.0001). The inhibitory effect of the anti–Activin A antibody decreased upon antibody dilution (Figure 3B).

At day 20, hiPSC-CMs were assessed at their intrinsic beat rate or paced at 1 Hz. When paced at 1 Hz, 1 nM Activin A reduced the impedance contractile amplitude (6.46 ± 0.23 vs 10.27 ± 0.36 ohms, *P* < 0.0001) and relaxation velocity kinetics (35.0 ± 1.1 vs 48.8 ± 2.7 ohms/s, *P* < 0.0001) compared with media control (Figure 3C). Anti–Activin A antibody (≥5 nM) prevented the Activin A–mediated reduction in impedance amplitude compared with cells exposed to Activin A plus control antibody (anti-Activin A 25 nM: 10.8 ± 0.99 vs 6.72 ± 0.38 ohms/s, *P* < 0.0001). Similarly, cells exposed to Activin A plus ≥ 5 nM anti–Activin A antibody had quicker relaxation velocity kinetics compared with cells exposed to Activin A plus control antibody (anti–Activin A 25 nM: 49.6 ± 4.0 vs 35.6 ± 2.7 ohms/s; *P* < 0.0001). Representative impedance traces are presented in Figure 3D.

### IL-1β induces Activin A expression in HCFs

**FIGURE 4:**
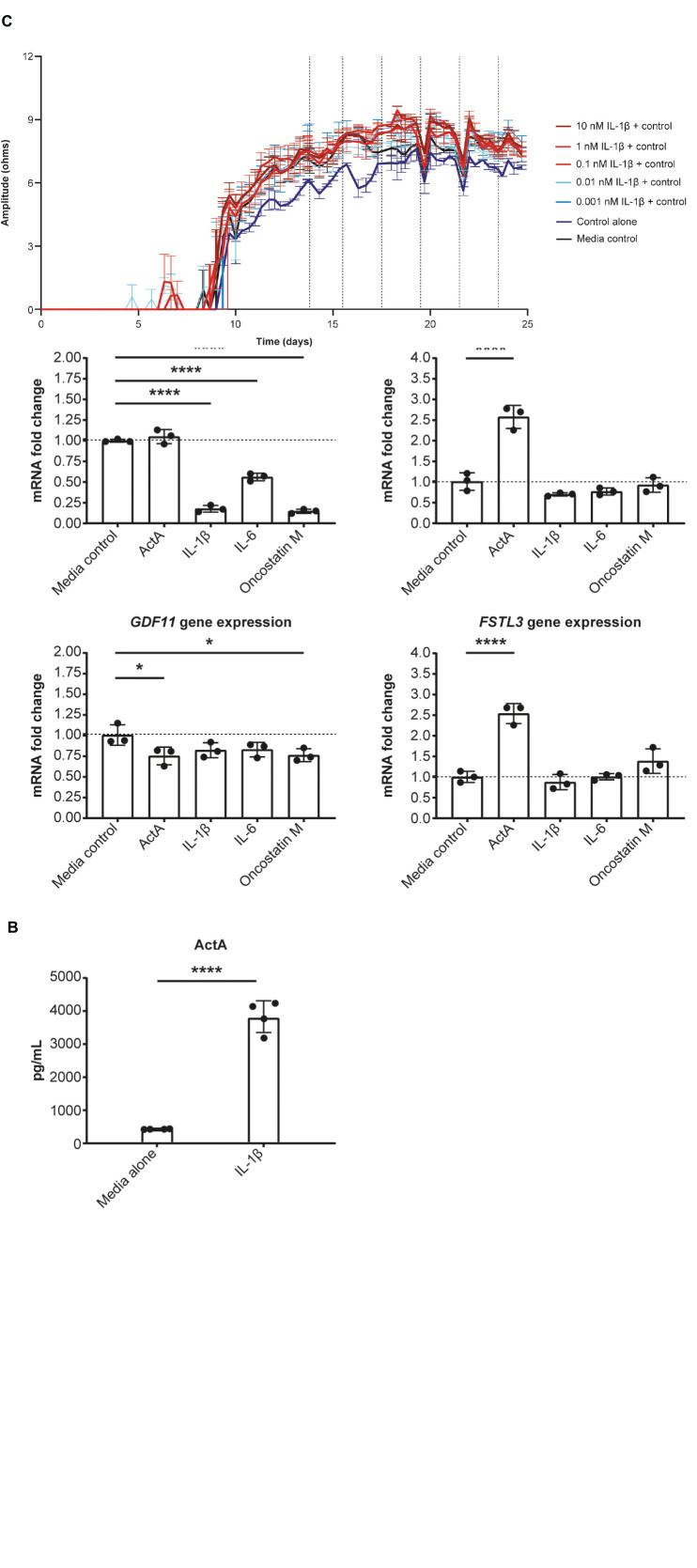
IL-1β induces Activin A gene expression in cardiac fibroblasts. **(A)** The effect of inflammatory cytokines, IL-1β, IL-6, and oncostatin M on gene expression in cardiac fibroblasts. **(B)** The concentration of Activin A in control- and IL-1β-treated cells. **(C)** The effect of IL-1β on induced pluripotent stem cell-derived CM contractility (vertical lines indicate media changes). One-way ANOVA was used to determine significance. ****P < 0.0001. ActA, Activin A; CM, cardiomyocyte.

Given our evidence that Activin A was detrimental for CM function, we tested whether pro-inflammatory cytokines present in failing human hearts contributes to local Activin A secretion in non-CMs. HCFs were treated with IL-1β, IL-6, oncostatin M, and Activin A. Expression of genes associated with activin and TGF-β signaling was then quantified using RT-qPCR (Figure 4A). Levels of *INHBA* mRNA were almost 4 times higher in cells treated with IL-1β compared with control cells (*P* < 0.0001). Treatment of HCFs with Activin A resulted in an approximately 2.5 times higher expression of *SERPINE1* and *FSTL3* (*P* < 0.0001), confirming the SMAD2/3 dependance of these genes in cardiac fibroblasts. Although Activin A had no effect on *MSTN* expression, it was reduced by ∼80% in cells treated with IL-1β or oncostatin M (*P* < 0.0001) and by ∼50% in cells treated with IL-6 (*P* < 0.0001). Significant reductions in *GDF11* expression were observed in cells exposed to oncostatin M (*P* < 0.05) and Activin A (*P* < 0.05), although expression was reduced (non-significantly) by all treatments.

Consistent with IL-1β–induced increase in expression of *INHBA* mRNA, the concentration of Activin A was approximately 9 times higher in the supernatant of HCFs treated with IL-1β than in that of control cells (Figure 4B). To investigate whether IL-1β is sufficient to recapitulate the impaired contractility induced by Activin A, contractile impedance amplitude was assessed in hiPSC-CMs exposed to IL-1β. IL-1β was found to have no direct effect on contractile impedance amplitude (Figure 4C). Similarly, IL-6 has no direct impact on CM function (data not shown). As TGF-β1 gene expression was increased with chronic treatment, we tested the impact of a pan-TGF-β inhibitory antibody on activin A-induced CM dysfunction. No direct impact on CM function was observed with anti-TGF-β (up to 30 nM) suggesting that TGF-β is not involved in the observed activin A-induced CM dysfunction (Supplementary Figure 1).

### Engaged Activin A signaling in hiPSC-CMs downregulates calcium cycling genes while promoting fibrotic gene expression

**FIGURE 5:**
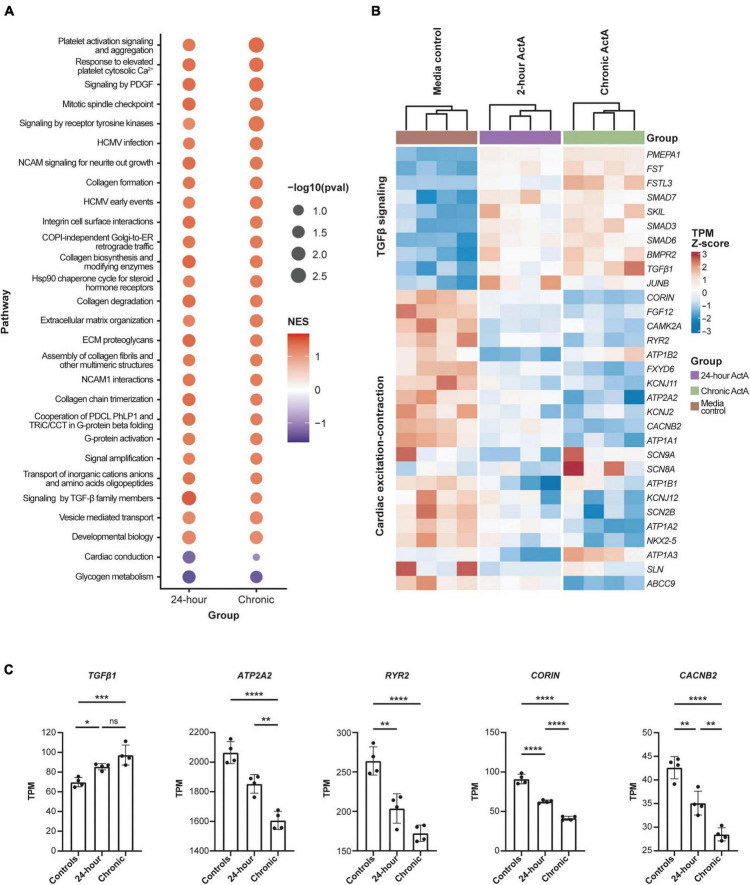
Activin A–dependent gene signature in hiPSC-CMs. **(A)** Significantly enriched pathways in hiPSC-CMs treated with acute (24 h) and chronic Activin A. All pathways were upregulated with positive normalized enrichment scores except glycogen metabolism and cardiac conduction. Cardiac conduction and signaling by TGF-β family members were only significant (*P* < 0.05) at 24 h. **(B)** Heatmap of leading-edge gene expression in cardiac conduction and signaling by TGF-β family members. Although both pathways were only significant after acute Activin A exposure (24 h), a similar pattern of gene expression was observed in chronic exposure. **(C)** TPM values for a subset of cardiac genes highlighted in the heat map. One-way ANOVA was used to determine significance. *P < 0.05, **P < 0.01, ***P < 0.001, ****P < 0.0001. ActA, Activin A; hiPSC-CM, human-induced pluripotent stem cell-derived cardiomyocyte; ns, not significant; TGF-β, transforming growth factor-β; TPM, transcripts per million.

To elucidate the transcriptional networks that lead to Activin A–mediated CM dysfunction, RNA sequencing was performed after acute (24 h) and chronic (6 repeated doses) Activin A exposure. Analysis of differentially expressed genes identified several pathways that were altered by exposure of hiPSC-CMs to Activin A (Figure 5A). Of the pathways that were significantly enriched following treatment with acute or chronic Activin A, all were upregulated with a positive normalized enrichment score, except for glycogen metabolism and cardiac conduction. Compared with control, signaling by TGF-β pathways was significantly (*P* < 0.05) upregulated with acute Activin A treatment. Figure 5B shows leading-edge genes extracted from GSEA. The 10 most up-regulated TGF-β signaling genes and the 21 most down-regulated genes involved in calcium cycling, electrophysiology, and contraction after chronic administration of Activin A are plotted. Note that, although the genes were selected based on perturbation in the chronic condition, these genes show consistent regulation in the acute condition as well.

Taking a candidate gene approach, differences for individual genes were present after both acute and chronic Activin A treatment. Chronic Activin A exposure caused upregulation of *TGF*-β*1* (∼39% higher compared with control, *P* < 0.001; Figure 5C). Chronic Activin A exposure caused significant downregulation, compared with control, of the critical calcium-handling genes *ATP2A2* (∼22%, *P* < 0.0001) and *RYR2* (∼35%, *P* < 0.0001), encoding sarcoplasmic/endoplasmic reticulum calcium ATPase 2 and ryanodine receptor 2, respectively. Additionally, expression of *CACNB2*, coding for the voltage-dependent L-type calcium channel subunit beta-2, and *CORIN*, coding for atrial natriuretic peptide-converting enzyme, were reduced by ∼33% (*P* < 0.0001) and ∼55% (*P* < 0.0001), respectively (Figure 5C). These observations suggest that Activin A–mediated signaling directly impacts genes important to coordinate normal CM conduction and contraction.

### Effect of Activin A on hiPSC-CM electrophysiology and calcium transients

**FIGURE 6:**
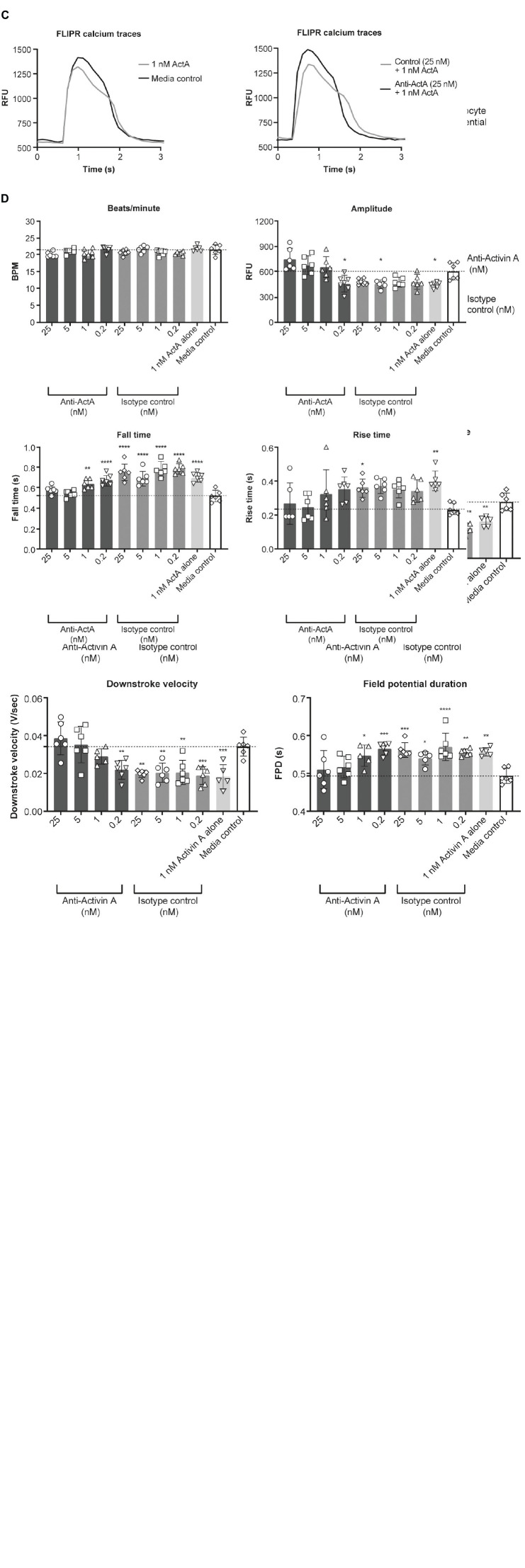
Effect of Activin A on myocardial electrophysiology and calcium transients. The effect of Activin A or Activin A plus inhibitory antibody on **(A,B)** electrophysiology and **(C,D)** calcium transients in induced pluripotent stem cell–derived cardiomyocytes. One-way ANOVA was used to determine significance. *P < 0.05, **P < 0.01, ***P < 0.001, ****P < 0.0001. ActA, Activin A; BPM, beats per minute; FLIPR, fluorescent imaging plate reader; FPD, field potential duration; IPSC, induced pluripotent stem cells; RFU, relative fluorescence units.

To determine the effect of Activin A on electrophysiology and calcium handling, hiPSC-CMs were assessed at their intrinsic beat rate after 6 Activin A doses at day 20. Elongated field potential duration was observed in hiPSC-CMs treated with chronic Activin A compared with control (0.56 ± 0.01 vs 0.49 ± 0.02 s, *P* < 0.01), which was prevented by 5 nM and 25 nM anti–Activin A antibody (0.51 ± 0.05 vs 0.56 ± 0.02 s with 25 nM control antibody; Figures 6A,B). Chronic Activin A exposure also resulted in a reduction in field potential amplitude and downstroke velocity compared with control (48.58 ± 6.52 vs 74.52 ± 11.66 μV, *P* < 0.01 and 0.018 ± 0.006 vs 0.034 ± 0.005 V/s, *P* < 0.001), respectively. Both of these were prevented by 25 nM anti–Activin A antibody (85.55 ± 19.82 vs 42.87 ± 2.00 μV with control antibody and 0.039 ± 0.009 vs 0.019 ± 0.002 V/s with control antibody), respectively (Figures 6A,B).

Calcium handling was analyzed to explore whether the mechanism of altered Activin A–induced electrophysiological changes in hiPSC-CMs was a result of impaired calcium flux (representative traces are displayed in Figure 6C). Chronic exposure with 1 nM Activin A reduced peak (max-min relative fluorescence units [RFU]) calcium amplitude (447 ± 33 vs 609 ± 99 RFU, *P* < 0.02 with control media), increased calcium falling time (0.70 ± 0.04 vs 0.52 ± 0.05 s, *P* < 0.0001), and increased calcium rising time (0.41 ± 0.06 vs 0.24 ± 0.04 s, *P* < 0.01; Figure 6D). Peak calcium handling amplitude was 751 ± 129 RFU in cells treated with Activin A plus anti–Activin A antibody (25 nM) compared with 484 ± 37 RFU in cells treated with Activin A plus control antibody. Slower calcium transient fall and rise times were also observed following chronic exposure to Activin A compared with Activin A plus inhibitory antibody (0.75 ± 0.08 vs 0.58 ± 0.04 s and 0.36 ± 0.05 vs 0.27 ± 0.1 s, respectively; Figure 6D).

### Effect of Activin A on arrhythmia and contractile function in HECTs

**FIGURE 7:**
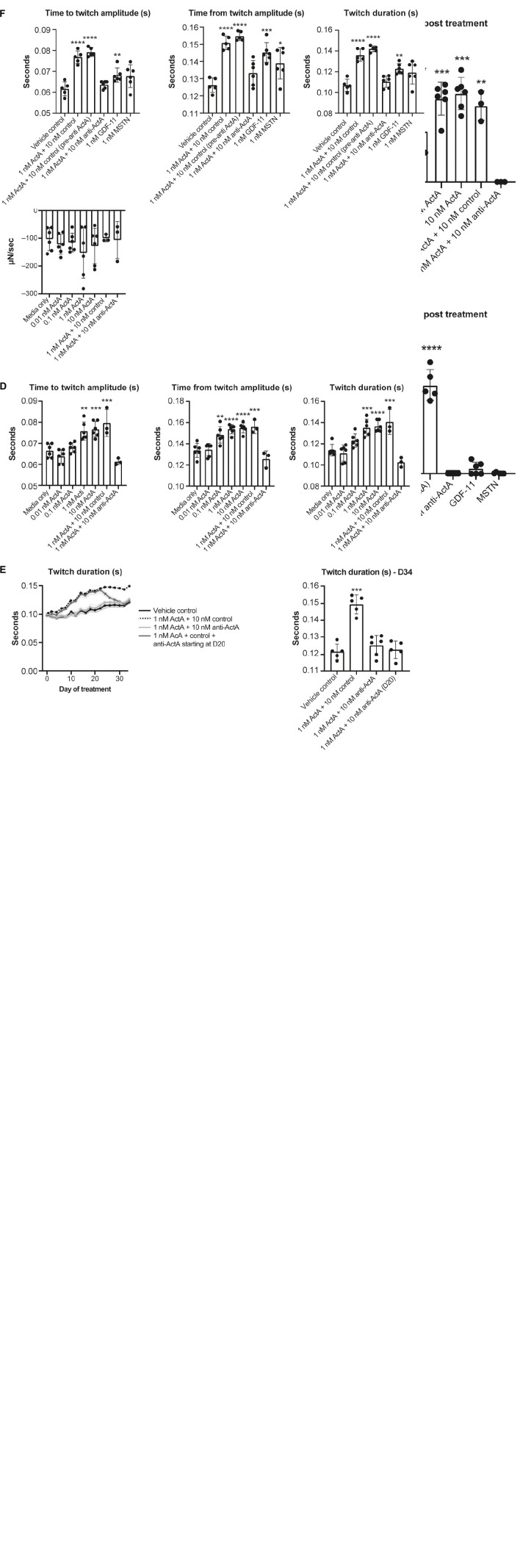
Effect of Activin A on contractile function and arrhythmia in HECTs. **(A)** Effect of Activin A on cardiac rhythm in HECTs for spontaneous beat rate (beating in the absence of pacing) during the full treatment period and at 8 days post treatment. **(B)** Effect of Activin A, MSTN (GDF8), and GDF11 on cardiac rhythm in HECTs for spontaneous beat rate during treatment and at 8 days post treatment. **(C,D)** impact of Activin A and preventive Activin A inhibition on HECT contraction kinetics on day 20 and the reversal by applying anti–Activin A starting on day 20 to day 34. **(E,F)** Impact of Activin A, Activin A inhibition, MSTN (GDF8), and GDF11 on HECT contraction kinetics on day 20. Shading on D denotes the 95% confidence interval. One-way ANOVA was used to determine significance. *P < 0.05, **P < 0.01, ***P < 0.001, ****P < 0.0001. ActA, Activin A; GDF, growth differentiation factor; HECT, human engineered cardiac tissue; MSTN, myostatin.

To better understand the contractile force and kinetics in a more mature model of human myocardium, a third model was studied using HECTs derived from hiPSC-CMs and HCFs. From a baseline level of 0% arrhythmic activity, chronic Activin A treatment caused a dose-dependent increase in arrhythmic activity over time, rising to 80% on day 8 and 67% on day 34 (24 doublet beats/30 total beats and 20 doublet beats/30 total beats, respectively; representative contraction curves in Supplementary Figure 2). Concurrent with (and likely underlying) the increased arrhythmic activity, there was an observed increase in spontaneous beat rate activity. This increase in spontaneous activity induced by 1 nM Activin A was prevented (treatment starting on day 1, Figures 7A,B) and reversed (treatment starting on day 20; Figure 7B) by 10 nM anti–Activin A antibody. At 8 days post-treatment (peak arrhythmic activity in this model), spontaneous beat rate was 0 Hz in HECTs treated with 1 nM Activin A and 10 nM anti–Activin A antibody, mirroring the control (Figures 7A,B). Interestingly, alternate TGF-β family members, myostatin (MSTN) (growth differentiation factor [GDF] 8) and GDF11 dosed at 1 nM failed to induce an increase in spontaneous activity (Figure 7B). Spontaneous beat rate was significantly faster in HECTs treated with 1 nM Activin A and 10 nM isotype control antibody compared with control (1.3 Hz vs 0.2 Hz; *P* < 0.0001) (Figure 7B).

There was no significant difference in twitch amplitude, maximum concentration slope, or maximum relaxation slope between all treatment conditions and media-only control on day 20 (Figure 7C). Consistent with our hiPSC-CMs, time to and from twitch amplitude and twitch duration increased in a dose-dependent manner following chronic treatment with Activin A on day 20 (Figure 7D). Mean time to twitch amplitude was significantly longer following treatment with 1 nM (*P* < 0.01) or 10 nM Activin A (*P* < 0.001) compared with control conditions. Similarly, mean time from twitch amplitude was significantly longer following treatment with 0.1 nM (*P* < 0.01), 1 nM (*P* < 0.0001), or 10 nM (*P* < 0.0001) Activin A, and mean twitch duration was significantly prolonged following treatment with 1 nM (*P* < 0.001) or 10 nM Activin A (*P* < 0.0001). Treatment with anti–Activin A, started at day 0, normalized twitch parameters, while the antibody isotype control had no effect (Figure 7D).

The effects of treatment with 1 nM Activin A on HECT spontaneous beat rate and twitch duration persisted for 34 days in the presence of 10 nM control antibody (Figures 7B,E). Importantly, these effects were suppressed in the presence of anti–Activin A and reversed when treatment was initiated on day 20 (Figure 7D). It is interesting to note that, at day 20, 1 nM MSTN (GDF8) and 1 nM GDF11 had intermediate effects when compared with Activin A on contractile kinetics compared with control (Figure 7F). It may be simply that Activin A is a more potent ligand than either GDF11 or MSTN; however, these findings support the contention that engaged Activin A signaling, and to a lesser extent GDF8 or GDF11, in CMs impairs cardiomyocyte contractile dynamics to promote dysfunctional myocardial performance.

## Discussion

The growing prevalence of heart failure among aging populations is a major concern (31) that has recently been exacerbated by the frequency of myocardial dysfunction in patients with COVID-19 (32, 33). IL signaling, SMAD2/3 phosphorylation, and Activin A signaling pathways have all been implicated in the development of cardiac dysfunction and age-related cardiovascular disease (15, 34–36). Recently, high levels of Activin A have also been found in patients with COVID-19, and are associated with poor outcomes and mortality (10, 11). Activin A may drive etiology-specific heart failure phenotypes, including cardioprotection from ischemic injury (37), while elevated circulating levels of Activin A are associated with cardiac dysfunction in non-ischemic heart failure mouse models as well as in humans (12, 15). The cell-type specific role of Activin A in the human heart is yet to be fully defined.

Here, we demonstrate that engagement of Activin A signaling is responsible for damping CM gene networks required to maintain optimal contractile function in the murine heart, hiPSC-CMs, and HECTs. In our Activin A over-expression mouse model (HDD), elevated circulating Activin A was sufficient to impair cardiac function in otherwise healthy young mice, confirming previous reports (15). Activin A caused increased phosphorylation of SMAD3 in CMs as well as significant upregulation of *SERPINE1* and *FSTL3*, markers of SMAD2/3 activation and activin signaling. Expression of genes associated with cardiac function, *ATP2A2, RYR2*, and *CACNB2* (associated with calcium dynamics); blood pressure and fluid balance (*CORIN* – processes natriuretic peptides); ion channel stability and function (*KCNJ2*, *SCN9A*, *SCN8A*, *SCN2B*); and protection from cardiac remodeling and heart failure (*KCNJ11*) were downregulated in hiPSC-CMs following Activin A exposure. Importantly, these same genes are known to be down-regulated in patients with heart failure and in experimental heart failure models (38–46).

We also observed prolonged calcium fall times following exposure to Activin A, which can be indicative of impaired CM diastolic function (47). The impairment of calcium channel kinetics was prevented by an anti–Activin A antibody. Downregulation of key calcium-handling genes *RYR2* and *ATP2A2*, induced by Activin A exposure, may contribute to the reduced peak calcium amplitude and increased calcium rise and fall times (48). Activin A–mediated upregulation of *FSTL3* and *SERPINE1*, downstream SMAD2/3 target genes, in our hiPSC-CMs showed that the activin receptor and its associated signaling are functionally intact in our model, and that Activin A can target hiPSC-CMs directly. These data lend support for Activin A in the upregulation of activin receptors following myocardial infarction, and is in line with previous results in rat models (14).

Inhibition of Activin A prevented and even reversed Activin A–induced CM dysfunction, suggesting that Activin A plays an important role in the observed pathologic effects. At the concentration tested (1 nM), neither GDF11 nor MSTN (GDF8) induced as robust a response as Activin A in the HECT model. These findings are consistent with other preclinical data supporting Activin A as the primary ligand driving cardiac dysfunction (15), as well as evidence showing that inhibition or disruption of the activin receptor type 2 signaling pathway improves cardiac function following experimental heart failure or myocardial infarction (15, 49). Inhibiting activin receptor type 2 and/or TGF-β receptor signaling following myocardial infarction may also improve calcium handling (49).

In both mice and humans, IL-1β and IL-6 are significantly upregulated following myocardial infarction and with advanced heart failure (50–53). In human primary cardiac fibroblasts, we found that IL-1β induced a strong upregulation of *INHBA*, the gene encoding the homodimeric subunits of Activin A. However, in contrast to published data collected in neonatal CMs (54), IL-1β had no direct effect on contractility in our hiPSC-CM assay. These data suggest that elevated levels of IL-1β in the serum of patients with heart failure or COVID-19 may act on cardiac fibroblasts to upregulate Activin A, which then directly impairs cardiac contractility. While COVID-19 was the not the initial focus of our work, recent clinical studies highlighted correlations between elevated Activin A levels and disease severity in COVID-19 (10, 11). In one preclinical study, hiPSC-CM treated with serum from patients with COVID-19 displayed marked arrhythmia (55). However, the arrhythmia was not abolished by adding the IL-1β inhibitor canakinumab to the culture. These data from Dimai et al. suggest that, while IL-1β is elevated in COVID-19, additional factor(s) are responsible for inducing arrhythmia (56). While based on our data this may be Activin A, it is important to note that levels of circulating TGF-β and Activin A have both been shown to correlate with disease severity in COVID-19 (15, 57). Since TGF-β and Activin A both induce SMAD3 phosphorylation and similar downstream signaling, the relative importance of Activin A– vs TGF-β–induced cardiac dysfunction in patients with COVID-19 requires further elucidation. Our data suggest that, in our hiPSC-CM model, activin induces CM dysfunction independent of TGF-β.

The results of this study show that Activin A directly impairs CM function and suggest that elevated levels of Activin A with aging, post COVID-19 infection, or after cardiac injury may directly contribute to cardiac dysfunction. Furthermore, we have identified a potential mechanism through which IL-1β may indirectly impair CM contractility through its upregulation of Activin A in cardiac fibroblasts (summarized in Supplementary Figure 3). This is the first study to demonstrate that Activin A acts directly on CMs, which may contribute to the cardiac dysfunction seen in aging populations, in patients with heart failure, and in patients with COVID-19–related morbidities.

## Data availability statement

Qualified researchers may request access to study documents and gene expression data that support the methods and findings reported in this manuscript. The RNAseq data (Figure 5) has been uploaded to NCBI. Submission ID: SUB12161861 and BioProject ID: PRJNA891053.

## Ethics statement

All animals received humane care in compliance with IACUC and the Principles of Laboratory Animal Care formulated by the National Society for Medical Research and the Guide for the Care and Use of Laboratory Animals prepared by the Institute of Laboratory Animal Resources and published by the National Institutes of Health (NIH publication 85–23, revised 1985).

## Author contributions

SM, JM, OZ, GH, MPM-P, MPG, and MED conceived and designed the research. JM, QR, DJ, KP, HE, XJ, DZ, JT, NTF, and AS performed the experiments. SM, JM, and QR analyzed the data. SM, JM, QR, OZ, GH, DJ, KP, NTF, MPG, MED, DG, and LM interpreted the results of experiments. SM, JM, OZ, and DJ prepared the figures. SM, JM, OZ, and GH drafted the manuscript. All authors edited and revised the manuscript and approved the final version of the manuscript.
