# Supplementary material for: Activin A directly impairs human cardiomyocyte contractile function indicating a potential role in heart failure development
Source: Front Cardiovasc Med. 2022 Nov 10;9:1038114. doi: 10.3389/fcvm.2022.1038114 (PMC9685658; doi:10.3389/fcvm.2022.1038114)
Supplement: Supplementary file 1 [file Data_Sheet_1.docx]

Supplementary Material

# Supplementary Figures and Tables

## Supplementary Table 1. TaqMan probes used for RT-qPCR analysis

| **Probe target (protein)** | **Assay ID** |
| --- | --- |
| GAPDH | Hs99999905_m1 |
| NPPA | HS0038323-_G1 |
| NPPB | HS00173590_M1 |
| ATPA2A | HS00544877_M1 |
| RYR2 | HS00181461_M1 |
| FSTL3 | HS00610505_M1 |
| SERPINE1 | HS06167155_M1 |
| INHBA | HS01081598_M1 |
| MSTN (GDF8) | Hs00976237_m1 |
| GDF11 | Hs00195156_m1 |

RT-qPCR, quantitative reverse transcription polymerase chain reaction.

## Supplementary Figure 1. The effect of chronic 1 nM Activin A and anti-TGF-β antibody on induced pluripotent stem cell-derived CM contractility (vertical lines indicate media changes)


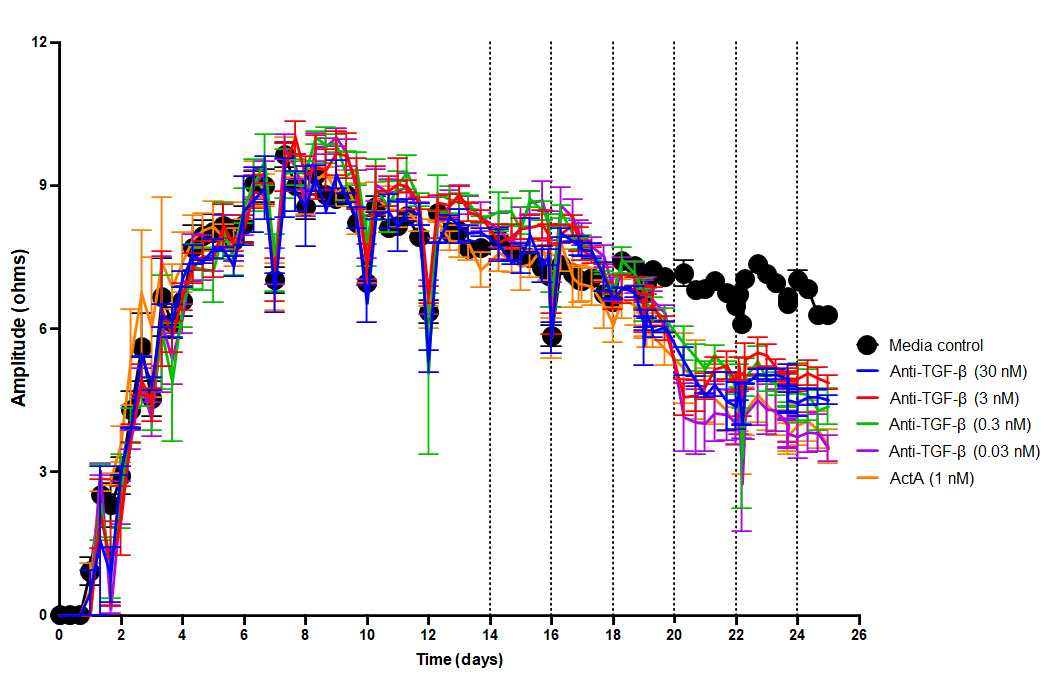


ActA, Activin A; CM, cardiomyocyte; TGF-β, transforming growth factor-β.

## Supplementary Figure 2. Impact of Activin A on arrhythmic activity in engineered cardiac tissue


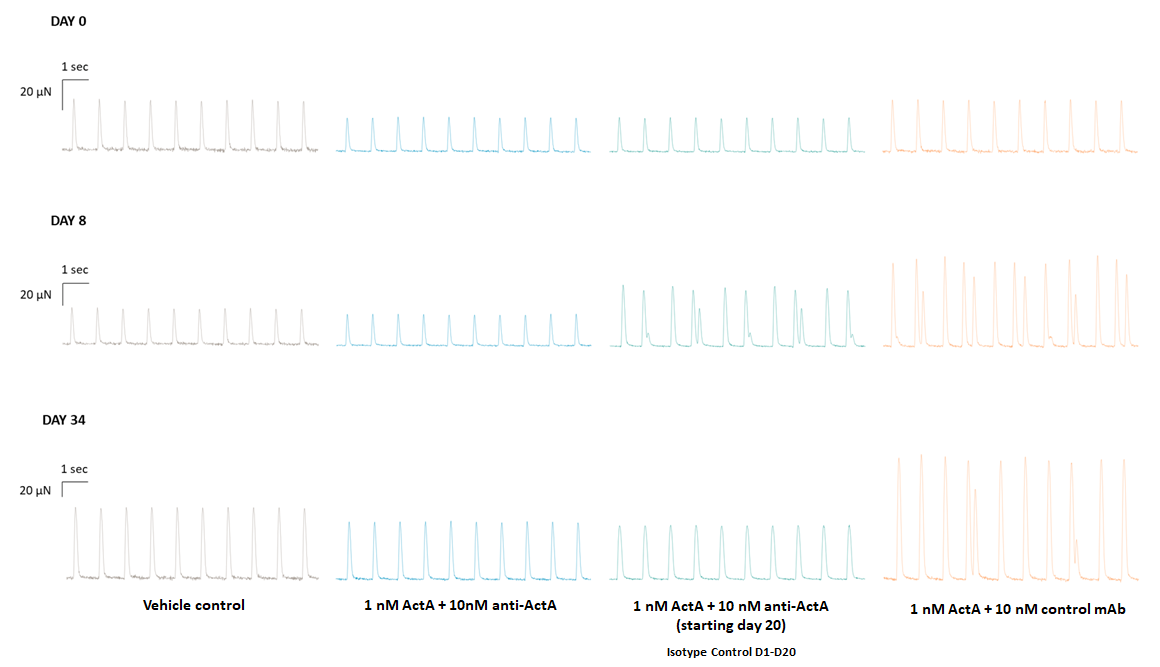


Functional data from human engineered cardiac tissue demonstrating increased arrhythmia with Activin A over time. When anti–Activin A is administered on day 20, the arrhythmia activity is reversed. ActA, Activin A.

## Supplementary Figure 3. Model demonstrating the potential mechanism through which cytokines may indirectly impair CM contractility through the upregulation of Activin A in cardiac fibroblasts


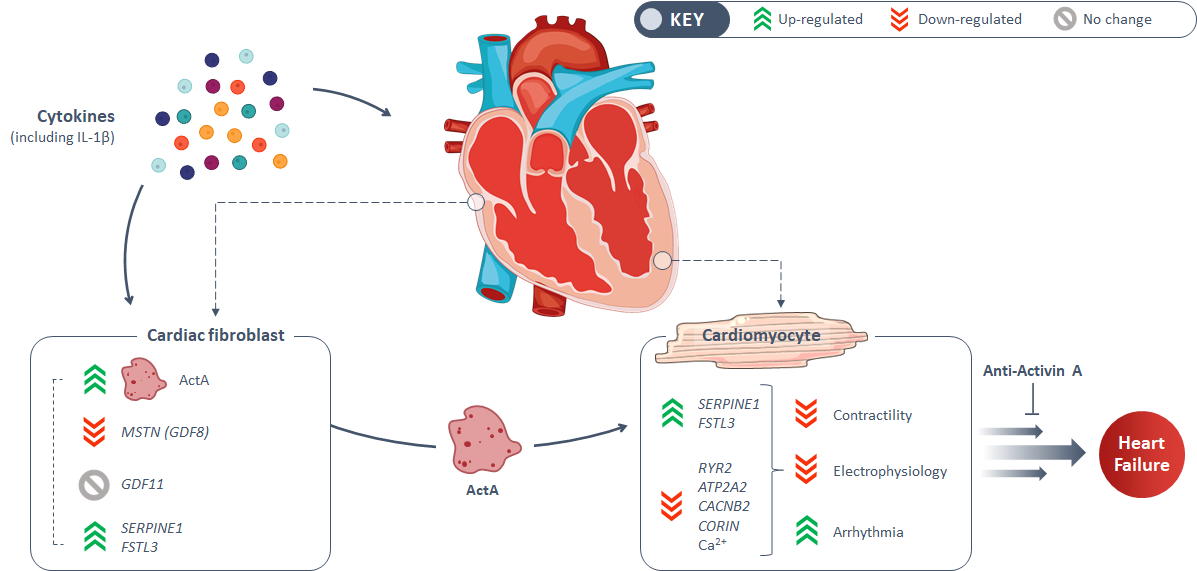


ActA, Activin A; CM, cardiomyocyte.
